# The effectiveness of protein supplementation combined with resistance exercise programs among community-dwelling older adults with sarcopenia: a systematic review and meta-analysis

**DOI:** 10.4178/epih.e2024030

**Published:** 2024-02-14

**Authors:** Phatcharaphon Whaikid, Noppawan Piaseu

**Affiliations:** Ramathibodi School of Nursing, Faculty of Medicine Ramathibodi Hospital, Mahidol University, Bangkok, Thailand

**Keywords:** Sarcopenia, Protein supplementation, Resistance exercise, Community, Meta-analysis

## Abstract

**OBJECTIVES:**

The combination of protein supplementation and resistance exercise shows promise for improving and maintaining muscle mass, strength, and performance in older adults with sarcopenia. This systematic review aimed to evaluate the effects of this combination on muscle mass, muscle strength, and physical performance in community-dwelling older adults with sarcopenia.

**METHODS:**

We conducted a comprehensive search of 4 electronic databases: PubMed, Scopus, Embase, and the MEDLINE Library. The search covered literature from January 2013 to January 2023 and followed the Preferred Reporting Items for Systematic Reviews and Meta-Analyses (PRISMA) guidelines. Two independent reviewers assessed the methodological quality of each study using the standard critical appraisal tool from the Joanna Briggs Institute (JBI). Meta-analysis was performed with the JBI Sumari program.

**RESULTS:**

The review included 7 randomized controlled trials and 1 quasi-experimental study, encompassing a total of 854 participants aged 60 years and above. The study durations ranged from 10 weeks to 24 weeks. An analysis of standardized mean differences (SMDs) showed that protein supplementation combined with resistance exercise significantly increased muscle mass (SMD, 0.95; 95% confidence interval [CI], 0.13 to 1.78; p<0.05) and muscle strength (SMD, 0.32; 95% CI, 0.08 to 0.56; p<0.05).

**CONCLUSIONS:**

Although the limited number of randomized controlled trials restricts the robustness of our conclusions, the evidence suggests that protein supplementation combined with resistance exercise is effective in enhancing muscle mass and strength in community-dwelling older adults with sarcopenia.

## GRAPHICAL ABSTRACT

[Fig f4-epih-46-e2024030]

## Key Message

Sarcopenia is a significant health concern. Given the contextual variations and the diverse factors that contribute to the prevalence of sarcopenia, delivering precision interventions to older adults diagnosed with sarcopenia who still reside in the community poses unique challenges. Therefore, precision interventions are vital for proper and feasible treatment planning, especially for early management actions, to reduce the impact of sarcopenia and its associated adverse effects in older adults. Our systematic review and meta-analysis showed that protein supplementation combined with resistance exercise is effective in enhancing muscle mass and strength in community-dwelling older adults with sarcopenia.

## INTRODUCTION

As individuals age, various physical and functional limitations in older adults become more evident [1]. The natural processes of aging notably affect skeletal muscle mass and strength [2]. Sarcopenia, a prevalent condition associated with aging, is characterized by the progressive loss of skeletal muscle mass and strength, which impacts physical performance [3]. There are 2 main categories of causative factors for sarcopenia: (1) primary sarcopenia, which is associated with natural aging, and (2) secondary sarcopenia, which is linked to factors such as inadequate physical activity, systemic diseases, drug use [4], and nutritional deficiencies [5]. Secondary sarcopenia can result from multiple contributing factors. Its consequences are significant, including a higher risk of falls [6], increased rates of hospitalization [7], elevated levels of cognitive impairment [8], reduced quality of life [9], a greater risk of mortality [10], and increased social and economic burdens for older adults and their families [11,12]. Studies from around the world indicate that the prevalence of sarcopenia in older adults varies depending on the population, setting, and diagnostic criteria used. In nursing homes, prevalence rates range from 38.1% to 85.0% [13-15], while in hospitals, the rates are between 22.6% and 23.0% [16,17]. In community settings, the prevalence is approximately 10%, which is lower than in other contexts [17].

Several evidence-based studies support the use of protein supplementation and resistance exercise for improving and maintaining skeletal muscle mass, strength, and performance in older adults with sarcopenia. These interventions show promising results without the need for pharmacological interventions. Resistance exercises, as endorsed in clinical guidelines [18], effectively prevent and reverse sarcopenia [19,20]. Protein supplementation has also demonstrated efficacy in preventing sarcopenia [21,22]. It is important to note that globally recommended protein intakes vary by age to support the necessary maintenance of muscle mass [23]. However, it is worth mentioning that many studies have reported inconclusive and unclear results regarding the effectiveness of protein supplementation alone in improving various components of sarcopenia [24].

Developing effective interventions for sarcopenia is crucial to reduce the disease burden and improve the overall well-being of the older population. Timely interventions are essential to delay adverse health outcomes, particularly those associated with sarcopenia. Although numerous systematic reviews and meta-analyses have explored the effects of exercise, nutrition, and their combination on sarcopenia in older adults, there is a noticeable research gap concerning the combination of protein supplementation with resistance exercise. This gap is especially evident in community-dwelling settings among older adults diagnosed with sarcopenia.

Existing worldwide systematic reviews often lack specificity, particularly when it comes to targeting community-dwelling older adults with sarcopenia [25,26]. Addressing this gap is crucial, as it underscores the need for targeted systematic reviews that can provide detailed insights into the effectiveness of combined interventions for this specific population. Given the contextual variations and the diverse factors that contribute to the prevalence of sarcopenia, delivering precision interventions to older adults diagnosed with sarcopenia who still reside in the community poses unique challenges. Therefore, precision interventions are vital for proper and feasible treatment planning, especially for early management actions, to reduce the impact of sarcopenia and its associated adverse effects in older adults.

This systematic review and meta-analysis aimed to evaluate the impact of combining protein supplementation with resistance exercise on muscle mass, strength, and physical performance in older adults diagnosed with sarcopenia in community settings. The results are expected to have implications for the incorporation of effective protein supplementation and resistance exercise regimens for community-dwelling older adults with sarcopenia.

## MATERIALS AND METHODS

### Data sources and searching strategy

This systematic review and meta-analysis adhered to the Preferred Reporting Items for Systematic Reviews and Meta-Analyses (PRISMA) statement. The protocol for this study was registered with PROSPERO under the registration number CRD42023429617.

The PICO (population, intervention, comparator or control, and outcome) framework was utilized to develop an accurate search strategy, as shown in Table 1. A systematic search was conducted of 4 databases (PubMed, Scopus, Embase, and the MEDLINE Library) for articles published between January 2013 and January 2023, without any language restrictions.

The search strategy was developed using predefined search terms, which included: (1) population, with terms such as “community-dwelling older adults,” “community-dwelling older persons,” “community dwelling elderly people,” “community-dwelling elderly,” and “community older people”; AND (2) intervention, which was further divided into (2.1) resistance exercise, encompassing “resistance training,” “multi-component exercise,” and “strength training”; AND (2.2) protein supplementation, including “nutrition supplement,” “milk supplement,” “amino acid,” “leucine,” and “beta-hydroxy-beta-methylbutyrate,” as well as combinations of keywords related to exercise and nutrition; AND (3) sarcopenia outcomes, further detailed as (3.1) muscle mass, with the term “skeletal muscle mass”; (3.2) muscle strength, using “handgrip” as a keyword; and (3.3) physical performance, with terms such as “5-chair stand,” “walking speed,” and “gait speed.” The most recent search of the electronic databases was conducted on January 31, 2023. The search was restricted to articles published in English and studies that involved older adults diagnosed with sarcopenia, including characteristics of both randomized controlled trials (RCTs) and non-RCTs.

### Inclusion/exclusion criteria and study selection

The eligibility criteria were as follows: (1) the article must be a full-text publication; (2) the study design should be an RCT or quasi-experimental; (3) participants should be older adults aged 60 years or above; (4) these older adults must be identified as having sarcopenia according to standard criteria; (5) the exercise interventions should consist of resistance training or a multi-component exercise regimen that includes aerobic, balance, and physical activity training; (6) the protein supplementation intervention must utilize protein sources such as whey protein, leucine, casein, milk, and soy, either in isolation or combined with other nutrients; (7) the study population should comprise community-dwelling older individuals; (8) the paper must be published in English.

The exclusion criteria for the study are as follows: (1) older adults with specific health conditions such as sarcopenia obesity, knee osteoarthritis, hip fracture, stroke, or non-communicable diseases, including diabetes, chronic kidney disease, chronic obstructive pulmonary disease, and cardiovascular diseases, as well as other clinical illnesses and characteristics typical of post-hospital older adults; (2) types of interventions that do not include resistance exercise and nutritional exercise, for example, medication and hormone therapy; (3) trials conducted *in vitro* or *in vivo* using animal models; (4) studies published as pilot studies, conference abstracts, or review articles.

### Data extraction and study quality

Two researchers independently extracted key data from the selected articles, which included: (1) the author’s name, (2) year of publication, (3) sarcopenia diagnosis criteria, (4) characteristics of the study population such as sex, age, and number of participants, (5) country or region, (6) details of the experimental intervention, including resistance exercise and protein supplementation, (7) duration and frequency of the intervention, and (8) components of sarcopenia assessed.

The credibility of the empirical evidence was independently assessed by 2 researchers using the Joanna Briggs Institute (JBI) critical appraisal checklists. These checklists include 9 items for quasi-experimental studies and 13 items for RCTs, which were used to evaluate the risk of bias. Each item was rated as “yes,” “no,” “unclear,” or “not applicable.”

### Statistical analysis

Data analysis was conducted using the JBI Sumari program. The required information for each study to utilize the software includes the sample size, means, and standard deviations (SDs). Additionally, the standard mean difference (SMD) should be selected when differences are present among the studies in the measurement tools used. The outcomes for muscle mass, strength, and physical performance were calculated using 95% confidence intervals (CIs). The heterogeneity of the study was assessed utilizing I^2^ statistics, categorized as follows: (1) might not be important (I^2^ = 0.0-24.9%), (2) may represent moderate heterogeneity (I^2^ = 25.0-49.9%), (3) may represent substantial heterogeneity (I^2^ = 50.0-74.9%), and (4) considerable heterogeneity (I^2^ = 75.0-100%). Furthermore, χ^2^ p-values < 0.1 were considered to indicate the presence of heterogeneity.

### Ethics statement

This study constituted a systematic review and meta-analysis, employing secondary data and did not involve any human or animal interventions. As a result, ethical approval was not necessary.

## RESULTS

**Figure 1.**
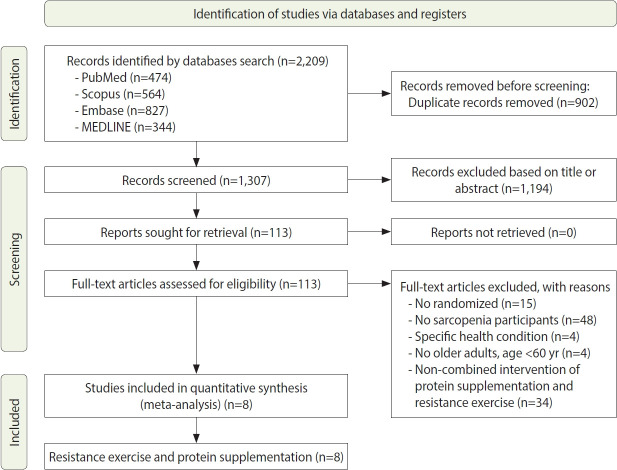
Preferred Reporting Items for Systematic Reviews and Meta-Analyses (PRISMA) diagram showing selection of study for systematic review and meta-analysis.

The process of the systematic review and meta-analysis is depicted in Figure 1, which follows the PRISMA flow diagram. We identified a total of 2,209 titles from various databases, including 474 articles from PubMed, 564 from Scopus, 827 from Embase, and 344 from MEDLINE, using specific search keywords. From this initial pool, 902 duplicate records were removed. We then screened 1,307 studies by their titles and abstracts. The next phase involved a full-text screening of 113 studies that met our inclusion criteria, which required a randomized study design and participants with sarcopenia who were older adults aged over 60 years, without any specific health conditions. Through this selection process, 105 studies were excluded from the meta-analysis. Ultimately, 8 articles were included in the quantitative synthesis.

### Study characteristics

This systematic review and meta-analysis included 8 articles that presented detailed characteristics of the studies, with a total sample of 854 older adults, as summarized in Table 2. The sample sizes of the included studies varied, ranging from 26 participants to 241 participants. Five of the studies included both male and female participants [27-31], while the remaining 3 studies exclusively involved male participants [32-34].

The studies included in this systematic review and meta-analysis were conducted across various regions: 6 in Asia, with 2 from China [27,31], and 1 each from Japan [28], Taiwan [32], Hong Kong [34], and Malaysia [29]. Additionally, there was 1 study from North America, specifically Canada [33], and 1 from Europe, in Sweden [30]. The duration of interventions in these studies ranged from 10 weeks to 24 weeks, with 5 studies lasting 12 weeks, 1 study lasting 24 weeks, 1 study lasting 16 weeks, and 1 study lasting 10 weeks. Furthermore, all studies incorporated a combination of resistance exercise and nutritional supplementation. The types of nutritional supplements used included whey protein, vitamin D, essential amino acids, milk, soy milk, soy protein, and ensure. The resistance exercise regimens included both pure resistance exercises and mixed exercises that combined resistance training with aerobic activities, as detailed in Table 2.

### Meta-analysis

This systematic review and meta-analysis examined 3 distinct outcomes: muscle mass, with a focus on appendicular skeletal muscle mass measured in kg/m^2^; muscle strength, with an emphasis on handgrip strength; and physical performance, concentrating on gait speed and the time to complete 5 chair stands.

#### Effects of protein supplementation combined with resistance exercise on muscle mass

**Figure 2.**
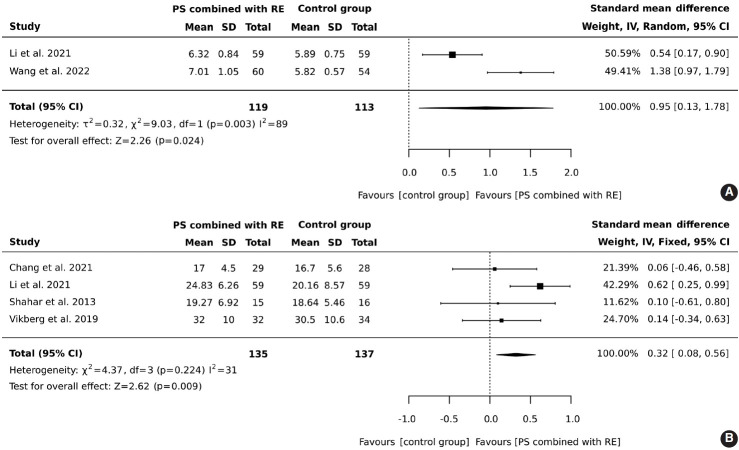
Forest plot of protein supplementation (PS) combined with resistance exercise (RE) on (A) muscle mass and (B) muscle strength in older adults aged 60 years and above. SD, standard deviation; CI, confidence interval; df, degrees of freedom.

In total, 119 older adults participated in an intervention combining protein supplementation with resistance exercise, while 113 were assigned to a control group. The results demonstrated that the combined intervention of protein supplementation and resistance exercise significantly increased muscle mass, with an overall effect size (SMD) of 0.95 (95% CI, 0.13 to 1.78), heterogeneity I^2^ = 89%, and Z = 2.26 (p = 0.024), as illustrated in Figure 2A.

#### Effects of protein supplementation combined with resistance exercise on muscle strength (handgrip strength)

A total of 135 older adults participated in a regimen combining protein supplementation with resistance exercise interventions, while 137 were assigned to a control group. The results demonstrated that the combination of protein supplementation and resistance exercise significantly improved handgrip strength (SMD, 0.32; 95% CI, 0.08 to 0.56; I^2^ = 31%; overall effect: Z= 2.62; p= 0.009), as illustrated in Figure 2B.

#### Effects of protein supplementation combined with resistance exercise on physical performance: the 5-chair stand test

**Figure 3.**
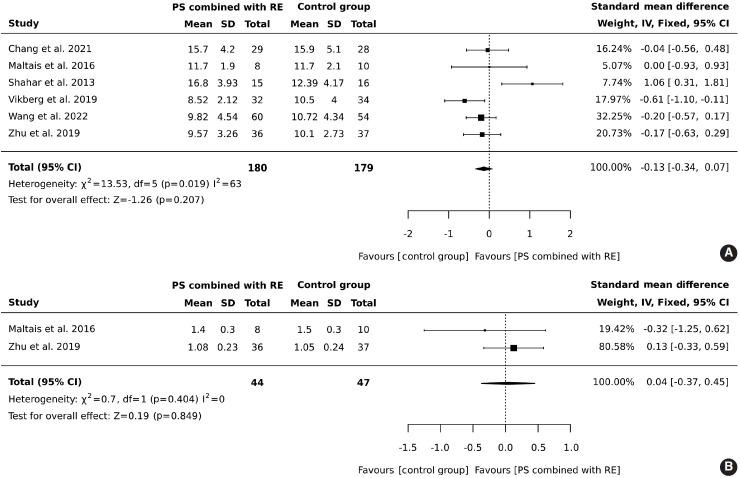
Forest plot of protein supplementation (PS) combined with resistance exercise (RE) on (A) physical performance (5-chair stand test) and (B) physical performance (gait speed) in older adults aged 60 years and above. SD, standard deviation; CI, confidence interval; df, degrees of freedom.

In total, 180 older adults participated in an intervention involving protein supplementation combined with resistance exercise, while 179 were assigned to a control group. The results indicated that the addition of protein supplementation to resistance exercise did not result in a significant improvement in the 5-chair stand test (SMD, -0.13; 95% CI, -0.34 to 0.07; I^2^ = 63%; overall effect: Z= -1.26; p= 0.207). Contrary to what might be expected, the control group outperformed the intervention group in the 5-chair stand test. However, this finding suggests that the combined intervention of protein supplementation and resistance exercise did not improve physical performance as measured by the 5-chair stand test compared to the control group, as shown in Figure 3A.

#### Effects of protein supplementation combined with resistance exercise on physical performance (gait speed)

A total of 44 older adults participated in combined protein supplementation and resistance exercise interventions, while 47 were part of the control group. The results revealed that the combination of protein supplementation and resistance exercise did not significantly increase gait speed (SMD, 0.04; 95% CI, -0.37 to 0.45; I^2^ = 0%; overall effect: Z= -0.19; p= 0.849). Furthermore, the comparison between the group receiving both protein supplementation and resistance exercise and the control group showed no significant improvement in physical performance, as depicted in Figure 3B.

### Publication bias and quality appraisal assessment

Upon critical appraisal using the JBI checklist for Quasi-Experimental and Randomized Controlled Trials, the evidence quality of the 8 studies included in this systematic review—1 quasi-experimental study and 7 RCTs—was determined.

The assessment indicated that the quasi-experimental study received a “yes” rating, while the RCTs were evaluated with a mix of “yes,” “no,” and “not applicable” responses. Within this group, 1 study had at least 2 “no” ratings, 2 studies each had at least 1 “not applicable” rating, and 1 study was marked with at least 2 “no” ratings and 2 “not applicable” ratings. Across all studies, key methodological aspects—randomization, allocation concealment, baseline comparability, completeness of follow-up, analysis consistency within randomized groups, uniformity of outcome measurement, reliability of outcomes, appropriateness of statistical analysis, and the adequacy of trial design—were all deemed to have a “low risk” of bias, achieving a 100% rating, as shown in Table 3.

## DISCUSSION

This systematic review and meta-analysis investigated the effect of protein supplementation combined with resistance exercise on muscle mass, muscle strength, and physical performance in community-dwelling older adults with sarcopenia. While the combination of protein supplementation and resistance exercise significantly improved sarcopenia, it may not fully address every aspect of the condition.

### Muscle mass

This systematic review and meta-analysis demonstrated that combining protein supplementation with resistance exercise significantly increased muscle mass. These results align with prior research indicating that increased protein supplementation is an effective method for augmenting muscle mass [35,36]. This is particularly true when the supplementation included leucine-enriched protein at doses of 1.2-6.0 g of leucine per day [36] or β-hydroxy β-methyl butyrate at 2-3 g/day [37]. Higher protein intake has been linked to greater increases in protein synthesis among older adults [37,38]. Additionally, resistance exercise is recognized as a key factor in boosting protein synthesis in skeletal muscle by activating the mechanistic target of rapamycin signaling pathway [39]. Resistance exercise is also recommended for its potential to prevent the loss of muscle mass and maintain it in older adults [40].

Consistent with previous meta-analyses, protein supplementation in combination with resistance exercise demonstrated the ability to significantly reverse muscle loss in older adults [25]. This finding is in agreement with an earlier study that showed a combined regimen of protein supplementation and resistance exercise had a more pronounced effect on increasing muscle mass than resistance exercise alone [25]. Earlier research has shown that skeletal muscle protein synthesis is significantly greater when highquality protein is consumed post-exercise compared to resistance exercise without supplementation [41], underscoring the role of protein supplementation in promoting muscle growth. However, despite previous findings, the effectiveness of protein supplements in increasing muscle mass remains inconclusive [36,42].

### Muscle strength

This systematic review and meta-analysis found that combining protein supplementation with resistance exercise significantly enhances muscle strength. These findings align with previous research, which suggested that a protein intake of 30 g to 45 g across 1-2 meals daily can lead to increased and sustained muscle strength [43]. Resistance exercise alters and rebuilds muscle fibers [44], leading to a marked increase in the size of both type 1 and type 2 muscle fibers and an elevation in muscle protein synthesis [45]. Additionally, it improves the generation of neuronal activity, thereby boosting muscle strength [44]. A critical element in this process is the plasticity of the neuromuscular system, which plays a significant role in the enhancement of muscle strength [46,47].

Conversely, combining protein supplementation with resistance exercise resulted in a more pronounced increase in muscle strength compared to resistance exercise alone [48]. Contrary to previous studies, our findings showed similarities between the 2 methods, and Beckwée et al. [49] also recommended the use of resistance exercise alone. However, without resistance exercise, protein supplementation did not confer benefits in enhancing muscle strength [50].

### Physical performance

In this study, the combination of protein supplementation with resistance exercise did not significantly improve physical performance, specifically in terms of gait speed. Similar studies have reported no significant changes in gait speed following resistance training [51], underscoring the limited effect of this type of training on that particular outcome. Furthermore, protein supplements alone did not result in improvements in gait speed [52], which points to the various factors that can influence outcomes across different populations. A previous systematic review and meta-analysis found that adding protein supplementation to resistance exercise did not significantly outperform resistance exercise alone [53,54]. This is consistent with research showing no difference in outcomes among older adults who received either intervention [55].

On the other hand, some studies have shown that combining protein supplementation with resistance exercise is more effective than standard care, especially when measured by the 5-chair stand test. This finding is consistent with prior research that has identified this combination as an effective method for improving physical performance, particularly with certain types of protein supplements [56]. However, these studies did not compare the effects of protein supplementation combined with exercise to the effects of protein supplementation alone. Consequently, because only a limited number of studies have made this direct comparison, it is difficult to determine the precise impact on physical performance.

However, this review has several limitations. First, there was considerable variation in the protocols for protein supplementation and resistance exercise interventions across the studies, including differences in types, delivery methods, doses, and durations. This variation makes it difficult to identify the most effective protocols. Additionally, the criteria used to diagnose sarcopenia were not consistent among the studies, which introduces the possibility that different components of protein supplementation may contribute to the observed improvements in sarcopenia in older adults. There were also inconsistencies in the outcome measurements due to the use of various assessment tools. For example, some studies utilized dual-energy X-ray absorptiometry, while others relied on bioelectric impedance analysis or computed tomography. The involvement of trained research assistants also varied, affecting the accuracy of outcome measurements. Some studies detailed formal training requirements for these assistants, whereas others did not enforce such standards. These variations must be taken into account for a thorough interpretation of the study findings.

## CONCLUSION

This systematic review and meta-analysis revealed a broad spectrum of protein supplementation strategies paired with resistance exercise interventions among community-dwelling older adults with sarcopenia. Our review underscores the efficacy of this combination—protein supplementation and resistance exercise—as the most beneficial intervention. Moreover, it is crucial to examine the effects of resistance exercise interventions in older adults with sarcopenia, with particular emphasis on the stage of the condition. Such targeted focus is vital for identifying the optimal and most effective approach to using protein supplementation in conjunction with resistance exercise for community-dwelling older adults with sarcopenia.

## Figures and Tables

**Figure f4-epih-46-e2024030:**
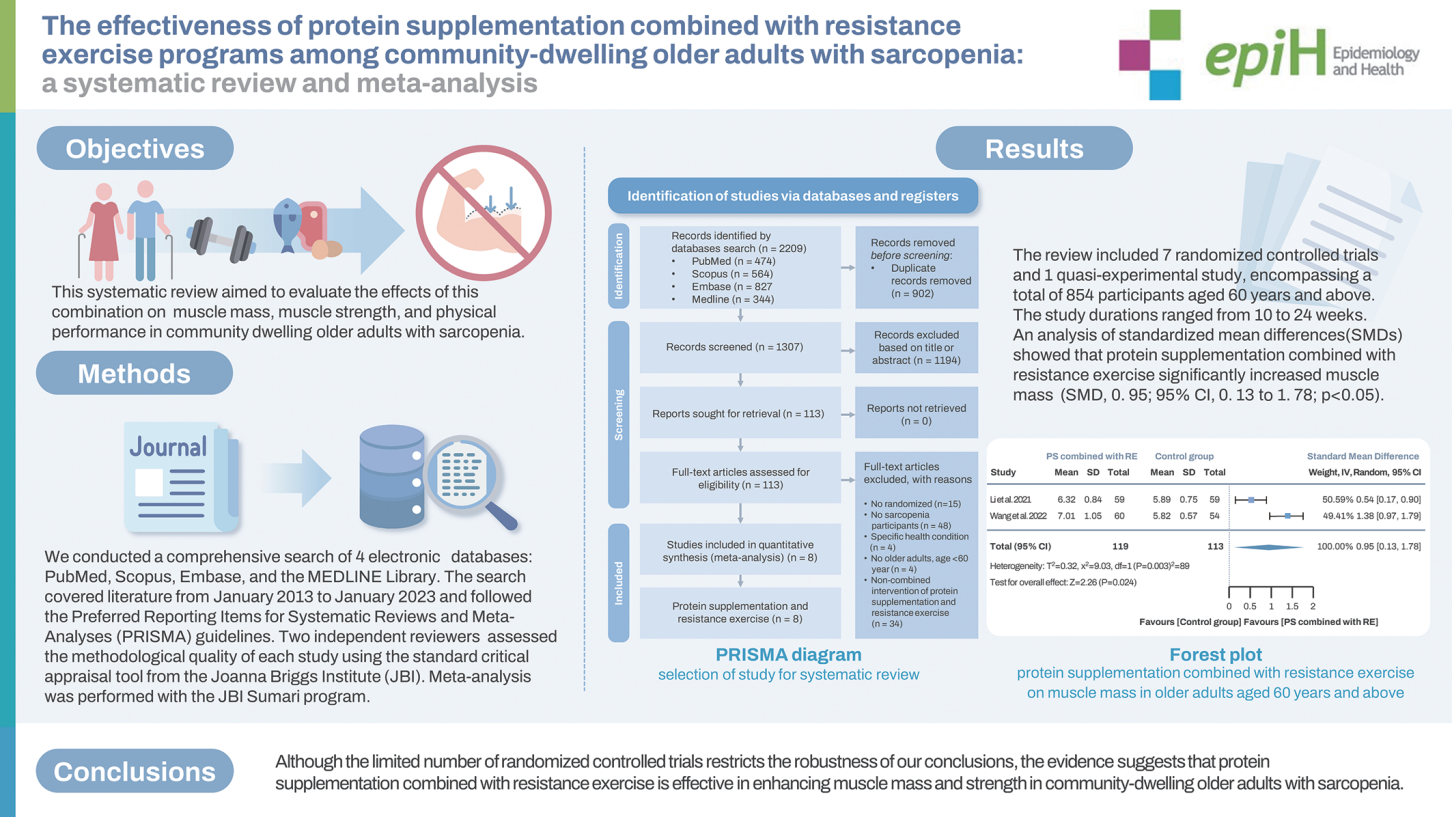


**Table 1.** PICO criteria for study inclusion

Parameters	Inclusion criteria
P: Participants	Worldwide community-dwelling older adults aged 60 yr or above with sarcopenia
I: Intervention	Underwent protein supplementation combined with resistance exercise
C: Comparison intervention	Usual care
Q: Outcome measures	Components of sarcopenia (muscle mass, muscle strength, physical performance)

**Table 2.** Characteristics of studies included in this systematic review and meta-analysis

Study	Diagnosis of sarcopenia	Participants (n)			Country study	Intervention		Duration (wk)	Component of sarcopenia
		Age (yr)	PS and RE	CG		RE	PS		
Shahar et al., 2013 [29]	BIA: SM cut-off points <0.75 kg/m^2^ for male and 6.75 kg/m^2^ for female	65 older adults with sarcopenia, age ≥60	15	16	Malaysia	RE program, 2 session/wk, 60 min for each session (elastic band)	One time/day	12	Improved
							- Soy protein drink 20 g/day and 40 g/day, in a powder form was given to male and female subjects, respectively		• Muscle mass
Maltais et al., 2016 [33]	MMI	26 older male with sarcopenia, age ≥60	8	10	Canada	RE program, 3 session/wk, 1-hr for each session (free weightlifting and resistance equipment for leg press, bench press, leg extension, and shoulder press, sit-ups, rowing extensions, biceps curls, and leg curls)	Suppl immediately after the exercise session	16	Improved
							1. EAA supp (12 g protein, 7 g of EAA)		• Muscle strength
							2. Milk supp (13.53 g protein, 7 g of EAA)		
Zhu et al., 2019 [34]	AWGS, 2014	113 older male with sarcopenia, age ≥65	36	37	Hong Kong	RE program, 2 session/wk, 90 min for each session (chair-based RE exercises, aerobic exercises); One-home session weekly	2 Sachets a day	12	Improved
							- Ensure NutriVigor daily from baseline to 12 wk (54.1 g powder; 8.61 g protein, 1.21 g BMD, 130 IU vitamin D and 0.29 g omega-3 fatty acid)		• Muscle strength
									• Physical performance
Vikberg et al., 2019 [30]	EWGSOP, 2010	70 older adults with sarcopenia, age ≥70	32	34	Sweden	RE program, 3 session/wk, 45 min for each session (body weight, suspension band)	One time/day	10	Improved
							- Week 1 to 7: milk 250 mL (21 g protein)		• Muscle mass
							- Week 8 to 10: milk 250 mL (30 g protein)		• Maintaining functional strength
Chang et al., 2021 [32]	EWGSOP, 2010	57 older male with sarcopenia, age ≥65	29	28	Taiwan	2 session/wk	1. 2 Sticks of daily branched-chain amino acids (BCAA-Amino Vital Pro^®^, Ajinomoto) (800 IU of cholecalciferol and 600 mg of calcium)	12	Improved
						1. A hospital-based program involving physical therapy and rehabilitation in hospital			• Muscle mass
						2. Home-based exercise (Keiser Sports Health Equipment)	2. 2 Tablets daily of calcium and vitamin D_3_ supplement (Caltrate, Pfizer, USA) for 12 wk (800 IU of cholecalciferol and 600 mg of calcium)		
Li et al., 2021 [27]	AWGS, 2014	241 older adults, age ≥60	59	59	China	Aerobic and RE program, 3 session/wk, 30 min for each session (dumbbells and sandbags)	1. PRO powder 10 g, 3 times daily with meals	12	Improved
							2. EPA (300 mg), DHA (200 mg), and vitamin D_3_ (250 IU) 2 pills/time and 2 time/day		• Muscle mass
									• Muscle strength
Wang et al., 2022 [31]	AWGS, 2019	201 older adults with sarcopenia, age ≥65	60	54	China	The app evaluated the participant’s exercise status and recommended the amount of exercise, such as 40 to 60 min of moderate-to-high-intensity exercise (brisk walking and jogging) and resistance training (seated leg raises, static squat against a wall, dumbbell lifts, elastic bands, etc.) for 30 min, ≥3 day/wk	The app featured the ability to assess each participant’s diet and provide recommendations for adjustments, focusing on energy and protein intake, especially the high-quality protein, and give recommended recipes	12	Improved
									• Muscle mass
Mori et al., 2022 [28]	AWGS, 2014	81 older adults with sarcopenia, age ≥65	21	21	Japan	RE program 2 session/wk, 40 min for each session	PRO supplement: 11.0 g of protein, and 2,300 mg of leucine per serving	24	Improved
							1. RT+PRO group ingested the protein supplement within 5 min after completion of the RT program		• Muscle mass
							2. PRO groups ingested the PRO supplement 3 hr after lunch		• Muscle strength

PS and RE, protein supplementation combined with resistance exercise; CG, control group; BIA, bioelectrical impedance analysis; SM, skeletal muscle; MMI, muscle mass index; EWGSOP, European Working Group on Sarcopenia in Older People; AWGS, Asian Working Group for Sarcopenia; DHA, docosahexaenoic acid; EPA, eicosapentaenoic acid; EAA supp, essential amino acid; Milk supp, milk supplement; RT, resistance training; PRO, whey protein.

**Table 3.** Joanna Briggs Institute (JBI) critical appraisal

Study	JBI critical appraisal of the eligible													
	Q1	Q2	Q3	Q4	Q5	Q6	Q7	Q8	Q9	Q10	Q11	Q12	Q13	Total score
Quasi-experimental study^1^														
Shahar et al. [29]	Y	Y	Y	Y	Y	Y	Y	Y	Y	-	-	-	-	9/9
Randomized controlled trials (RCTs)^2^														
Maltais et al. [33]	Y	Y	Y	Y	Y	Y	Y	Y	Y	Y	Y	Y	Y	13/13
Zhu et al. [34]	Y	Y	Y	Y	N	N	N/A	Y	Y	Y	Y	Y	Y	10/13
Vikberg et al. [30]	Y	Y	Y	Y	Y	Y	Y	Y	Y	Y	Y	Y	Y	13/13
Chang et al. [32]	Y	Y	Y	Y	Y	Y	Y	Y	Y	Y	Y	Y	Y	13/13
Li et al. [27]	Y	Y	Y	N/A	N	N	N/A	Y	Y	Y	Y	Y	Y	9/13
Wang et al. [31]	Y	Y	Y	Y	N	N	Y	Y	Y	Y	Y	Y	Y	11/13
Mori et al. [28]	Y	Y	Y	Y	N	N	N/A	Y	Y	Y	Y	Y	Y	12/13

Y, yes; N, no; N/A, not applicable.

1 Q1: Is it clear in the study what is the ‘cause’ and what is the ‘effect’ (i.e., there is no confusion about which variable comes first)?; Q2: Were the participants included in any comparisons similar?; Q3: Were the participants included in any comparisons receiving similar treatment/care, other than the exposure or intervention of interest?; Q4: Was there a control group?; Q5: Were there multiple measurements of the outcome both pre and post the intervention/exposure?; Q6: Was follow up complete and if not, were differences between groups in terms of their follow up adequately described and analyzed?; Q7: Were the outcomes of participants included in any comparisons measured in the same way?; Q8: Were outcomes measured in a reliable way?; Q9: Was appropriate statistical analysis used?

2 Q1: Was true randomization used for assignment of participants to treatment groups?; Q2: Was allocation to treatment groups concealed?; Q3: Were treatment groups similar at the baseline?; Q4: Were participants blind to treatment assignment?; Q5: Were those delivering treatment blind to treatment assignment?; Q6: Were outcomes assessors blind to treatment assignment?; Q7: Were treatment groups treated identically other than the intervention of interest?; Q8: Was follow up complete and if not, were differences between groups in terms of their follow up adequately described and analyzed?; Q9: Were participants analyzed in the groups to which they were randomized?; Q10: Were outcomes measured in the same way for treatment groups?; Q11: Were outcomes measured in a reliable way?; Q12: Was appropriate statistical analysis used?; Q13: Was the trial design appropriate, and any deviations from the standard RCT design (individual randomization, parallel groups) accounted for in the conduct and analysis of the trial?
